# Clinical and Sociodemographic Characteristics of Young People With Early Psychosis: Comparing Cannabis Users and Non‐Users in a Specialized Early Intervention Setting

**DOI:** 10.1111/eip.70240

**Published:** 2026-07-27

**Authors:** Anna Willert, Laura von Hardenberg, Laura Holzner, Michèle Kallenbach, Eva Reichert, Andreas Bechdolf, Karolina Leopold

**Affiliations:** ^1^ Department of Psychiatry, Psychotherapy and Psychosomatics Vivantes Klinikum Am Urban and Vivantes Klinikum Im Friedrichshain Berlin Germany; ^2^ Department of Psychiatry and Psychotherapy, Charité Campus Mitte Charité Universitätsmedizin Berlin Berlin Germany; ^3^ Deutsches Zentrum für Psychische Gesundheit (DZPG) Berlin‐Potsdam Germany; ^4^ Department of Psychiatry and Psychotherapy, University Hospital Carl Gustav Carus Technical University Dresden Dresden Germany

## Abstract

**Background:**

Despite the high prevalence of comorbid cannabis use at first onset of psychotic symptoms, high transition rates from cannabis‐induced psychosis to schizophreniform psychoses and bipolar disorder, and evidence of cannabis use as a risk factor in developing primary psychosis, only a few studies have addressed the difficulties in differential diagnosis focusing on the early stages of the disease.

**Aims:**

The present study is the first to date to examine clinical and sociodemographic characteristics of individuals with early psychosis, comparing cannabis‐induced psychotic disorder (CIPD), non‐substance‐induced psychotic disorder with concurrent cannabis use (NSIPD+C), and non‐substance‐induced psychotic disorder (NSIPD).

**Method:**

A retrospective chart review of patients aged 18–35 with early psychosis who received inpatient treatment at FRITZ Early Intervention Center between December 2016 and September 2021 was conducted. Sociodemographic characteristics included age, biological sex, family history of psychiatric illness, migration background, living status, education/work status. Clinical characteristics included insight into illness, duration of untreated psychosis (DUP), social functioning, pathway to treatment, treatment motivation, treatment recommendation, and type of discharge.

**Results:**

Of 509 participants, 42.6% had a diagnosis of NSIPD, 33.3% of CIPD, 7% of NSIPD+C. NSIPD had a significantly longer treatment duration compared to CIPD, a higher treatment motivation, and a higher discharge in accordance with clinicians' recommendation compared to both CIPD and NSIPD+C. No significant group differences were found in sociodemographic characteristics, DUP, and functioning scores.

**Conclusions:**

The examined sociodemographic parameters as well as level of functioning and DUP do not aid in the differential diagnostic classification when cannabis use and psychotic symptoms occur simultaneously. The absence of differences in DUP across diagnoses may reflect the efficacy of low‐threshold access provided by FRITZ and further supports the need for comprehensive implementation of early intervention services.

## 1 Introduction

Compared to other substance‐induced psychoses, cannabis‐induced psychotic disorders (CIPD) show the highest transition rates to schizophreniform psychoses or bipolar disorder at almost 50% (Murrie et al. 2020; Starzer et al. 2018). In addition, there is an increased risk of transitioning to a primary psychotic disorder within the first 3–5 years after diagnosis of CIPD, making the simultaneous occurrence of psychotic symptoms and cannabis use highly relevant in the early detection and intervention of psychotic disorders. Despite the very high prevalence up to 30%–50% of comorbid cannabis use at the first onset of psychotic symptoms (Kamali et al. 2009; Mauri et al. 2006; Wade et al. 2006) and the evidence of cannabis use as a risk factor for the development of schizophreniform psychoses (Di Forti et al. 2015, 2019), there are still only a few studies addressing the question of the extent to which a differential diagnosis of primary psychosis with concurrent cannabis use (NSIPD+C) and CIPD is possible in the early stages of the disease.

In first episode psychosis (FEP) cannabis use is linked to increased positive symptoms (Addington and Addington 2007; Grech et al. 2005; Ricci, Ceci, et al. 2023), less pronounced negative symptoms (Ricci, Ceci, et al. 2023), aberrant salience (Ricci, Di Muzio, et al. 2023), an earlier age of onset (Large et al. 2011; Seddon et al. 2016; Stone et al. 2014), poorer social functioning (Seddon et al. 2016), and a longer duration of untreated psychosis (DUP) (Schimmelmann et al. 2012; Seddon et al. 2016) with the latter representing a possible prognostic factor regarding the course of the illness (Howes et al. 2021).

Comparisons on the basis of diagnosis showed that patients with CIPD versus NSIPD+C presented with more pronounced anxious‐depressive symptoms (Rubio et al. 2012) and better performance in neuropsychological tasks requiring saccadic eye movements (Woolridge et al. 2023). Furthermore, the diagnosis of CIPD correlated with a better insight in relation to diagnosis (Rubio et al. 2012; Woolridge et al. 2023). In the first study comparing patients with primary psychosis without concurrent cannabis use (NSIPD), NSIPD+C, and CIPD, Rentero et al. demonstrated significant group differences between the CIPD cohort and the primary psychosis cohorts, regardless of concomitant cannabis use. Patients with CIPD presented more pronounced negative symptoms, less pronounced auditory hallucinations, and a higher incidence of manic symptoms (Rentero et al. 2021).

In summary, it should be noted that the findings to date provide a heterogeneous and inconclusive picture. Limitations in the scientific evidence arise from the fact that previous studies on this issue (a) examined small numbers of cases (Woolridge et al. 2023) and (b) also examined patients who did not meet early psychosis criteria and were therefore not in an early phase of a psychotic illness (Rentero et al. 2021; Rubio et al. 2012).

## 2 Methods

Data collection was performed retrospectively and based on clinical routine data. Therefore, no informed consent was obtained. The authors assert that all procedures contributing to this work comply with the ethical standards of the relevant national and institutional committees on human experimentation and with the *Helsinki Declaration* of 1975, as revised in 2013. All procedures involving human subjects/patients were approved by the Ethics Committee at Charité Universitätsmedizin Berlin on April 24, 2024 (EA1/078/24).

### 2.1 Study Design and Setting

This retrospective cohort study was conducted at FRITZ (an Early Intervention and Therapy Centre) in Berlin, Germany. The FRITZ intervention program was developed for young people with EP aged 16–35 years and offers in‐, outpatient and home treatment services (Bechdolf et al. 2021; Siebert et al. 2022). Only young people with EP who received inpatient or day clinic treatment were included in this study. Data was retrieved from December 1st, 2016, until December 1st, 2018, through a retrospective chart review. From December 1st, 2018, onwards, a standardized evaluation sheet was designed based on previous data collected through chart reviews. For patients starting inpatient or day clinic treatment between December 1st, 2018, and September 30th, 2021, data—including follow up—was collected directly from the treating clinicians using the evaluation sheets.

### 2.2 Participants

If admitted to the FRITZ inpatient or day clinic, participants were recruited between December 2016 and September 2021. Inclusion criteria were in accordance with Bird et al. (Bird et al. 2010): (1) diagnosis of a psychotic disorder, including cannabis‐induced psychosis F12.5, schizophrenia‐spectrum‐ disorders F2x, mania with psychotic symptoms F30.2, bipolar affective disorder with psychotic symptoms F31.2/F31.5, and severe depression with psychotic symptoms F32.3/F33.3 (2) onset of the first psychotic episode did not exceed 5 years, or presentation to mental health services did not occur more than 5 years ago, (3) 18–35 years old, and (4) a minimum inpatient or day clinic stay of 48 h. Although no formal IQ or cognitive tests were administered, participants with an ICD‐10 diagnosis of intellectual disability were excluded to ensure diagnostic clarity in this early psychosis sample. Despite the high prevalence of polysubstance use among patients in early intervention services, we did exclude all patients with a diagnosis of comorbid substance use according to ICD‐10 (F1x.1, F1x.2) other than cannabis use (F12.1, F12.2) to avert comparison with secondary psychosis induced by substances other than cannabis.

### 2.3 Measures

Data recording on the FRITZ ward differed by time period. For patients admitted between 2016 and 2018, sociodemographic and clinical information was documented exclusively in individual patient charts. In 2018, the FRITZ ward treatment team developed and implemented a standardized evaluation sheet that resembled the structure and content of individual patient charts and was used for routine clinical documentation 2018 onward.

For the purpose of this study, data from both sources—patient charts (2016–2018) and standardized evaluation sheets (2018–2021) —were retrieved retrospectively. Diagnoses were assigned by the treatment team specialized in psychotic disorders on the FRITZ ward. As diagnoses were made using ICD‐10, a conversion to DSM‐5 guidelines was performed and the results are presented in Table 1. Diagnosis of CIPD was, therefore, based on ICD‐10 criteria of psychotic symptoms occurring within 6 months of substance use. Each patient and their diagnosis were discussed at weekly medical rounds, which included the senior consultant, ward physicians, psychotherapists and clinical psychologists, and diagnoses were assigned only by consensus.

**TABLE 1:** ICD‐10 versus DSM‐V diagnoses.

Name of disorder	ICD‐10	DSM‐V	NSIPD, *n* (%)	CIPD, *n* (%)	NSIPD+C, *n* (%)
Paranoid schizophrenia	F20.0	295.90	109 (50.2)	0	21 (58.3)
Schizotypal disorder	F21	297.1	2 (0.9)	0	0
Schizoaffective disorder	F25	295.70	12 (5.5)	0	6 (16.7)
Delusional disorder	F22	297.1	5 (2.3)	0	0
Acute and transient psychotic disorder/brief psychotic disorder	F23	298.8	57 (26.3)	0	1 (2.8)
Unspecified psychosis not due to a substance or known physiological condition	F29	298.9	5 (2.3)	0	0
Bipolar disorder, current episode manic severe with psychotic features	F31.2	296.44	19 (8.8)	0	5 (13.9)
Bipolar affective disorder, current episode severe depression with psychotic symptoms	F31.5	296.54	4 (1.8)	0	2 (5.6)
Major depressive disorder, recurrent, severe with psychotic symptoms	F33.3	296.34	3 (1.4)	0	1 (2.8)
Cannabis‐induced psychotic Disorder	F12.5	0	0	173 (100)	0
Cannabis abuse	F12.1	305.20	0	0	36 (100)

Abbreviations: CIPD, Cannabis‐induced Psychotic Disorder; *N*, number of participants; NSIPD, non‐substance‐induced psychosis disorder; NSIPD+C, non‐substance‐induced psychosis disorder with cannabis abuse.

#### 2.3.1 Sociodemographic and Clinical Characteristics

Sociodemographic characteristics collected included age, biological sex, family history of psychiatric illness, migration background, living status, educational background, and working status.

Clinical characteristics included the following:

*Insight into illness* was assessed using a 3‐point Likert scale with the following categories: none, limited, and present. Clinicians rated participants' level of illness insight at baseline, following diagnostic confirmation, and again at discharge.

*Duration of untreated psychosis (DUP)* was measured by counting the number of days between fulfilling the diagnostic criteria for a psychotic disorder and the start of the first antipsychotic treatment.

*The Global Assessment of Functioning (GAF*) is a scale ranging from 0 to 100 (APA 1987). Clinicians use it to assess the severity of a mental illness and its effects on social and occupational functioning. The lower the score, the more severely ill an individual is. This study noted GAF on the day of admittance and at discharge.

*Pathway to Treatment* was assessed using an adjusted version of the Pathways to Care Encounter Form (Lincoln et al. 1998) and defined as the primary referral pathway leading to admission to the FRITZ ward. Pathways were coded as a categorical variable with seven predefined categories: emergency room, locked inpatient ward, early intervention consultation session, outpatient services, different hospital, child and adolescent hospital, and planned admission via outpatient clinicians' or direct patient contact.

*Treatment on FRITZ* was explored by assessing different treatment aspects: length of treatment in terms of days, antipsychotic monotherapy vs. polytherapy, prescription of antidepressants, therapy sessions attendance (always, often and never), and therapy motivation. Motivation for therapy was assessed by clinicians and categorized into three levels: high, medium, or low. These ratings were based on a comprehensive clinical evaluation that included individuals' willingness to engage in and actively participate in group therapy, their attitudes toward prescribed medication, and qualitative information obtained from individual therapy sessions, including expressed beliefs, concerns, and treatment goals.

*Treatment recommendations provided to the patients at time of discharge*: The recommendations reflected clinicians' recommendations regarding appropriate follow‐up care based on patients' clinical needs at discharge. Recommendations were coded as categorical variables indicating whether a given type of service was recommended (yes/no). Recommended services included FRITZ‐specific programs (FRITZ day clinic; FRITZ group therapies), other structured treatment settings (rehabilitation center, day clinic, specialized inpatient addiction wards), outpatient services (outpatient center, specialized outpatient addiction centers, or further treatment once discharged from the FRITZ ward included a FRITZ day clinic, FRITZ group therapies, outpatient psychiatrist, outpatient psychotherapist), and psychosocial support interventions (job coaching and family support groups). Multiple recommendations could be assigned to a single patient.

*Type of discharge*: This was measured by examining whether the discharge was against medical advice, on disciplinary grounds, or in accordance with the treating clinicians.

*Measurements of cannabis consumption*: Cannabis use was defined as regular use at least three times per week. At the time of data collection, no routine assessment of detailed cannabis use patterns was applied. Therefore, no detailed information, for example, on exact frequency or amount of use could be provided.

### 2.4 Analyses

Participants were divided into three groups for the study. The first group comprised participants diagnosed with psychosis (ICD 10: F20.0; NSIPD) who did not use any substances. The second group consisted of participants diagnosed with primary psychosis and cannabis abuse (ICD 10: F20.0 combined with F12.1; NSIPD+C). The third group included participants diagnosed with cannabis‐induced psychotic disorder who did not use any other substances (ICD 10: F12.5; CIPD). The study assessed the differences in sociodemographic and clinical characteristics among the three groups. All analyses were conducted using the SPSS software, and the tests were two‐sided with an alpha level of 0.05.

Change in functioning was assessed using GAF. For each individual, a GAF change score was calculated by subtracting the baseline GAF score (at baseline) from the GAF score at discharge. Positive change scores indicated improvement in overall functioning over the course of treatment, whereas negative scores indicated a decline in functioning.

As data for the continuous variables (age, DUP, length of treatment, and GAF score at discharge) were not normally distributed, we conducted a multivariate Kruskal–Wallis test. Post hoc comparisons were performed for each variable using Dunn Tests with a Bonferroni‐adjusted alpha level of 0.016. Chi‐squared tests were used to compare categorical variables. When expected cell counts were below 5 for the variables ‘type of discharge’, ’therapy participation’ and ’therapy motivation’, categories were combined to meet assumptions for statistical analysis. Specifically, discharges *against medical advice* and *on disciplinary groups* were combined. For therapy participation, the categories *never* and *often* were combined and compared with *always*. Similarly, for therapy motivation, *low* and *medium* were combined and compared with *high*. Post hoc comparisons were performed for each significant result using the Bonferroni adjustment (alpha level of 0.016).

For the variable *pathway to care*, one diagnostic subgroup (NSIPD+C) comprised a small number of cases. Consequently, analyses were restricted to the two most frequent pathways: emergency room admission and transfer from a locked inpatient psychiatric ward—each exceeding a sample size of five across groups.

## 3 Results

Between 2016 and 2021, 867 participants were screened for inclusion in the study. Individuals who did not meet the inclusion criteria (*n* = 37) and those with multiple admissions (*n* = 321), in which case only the first admission to FRITZ was considered, resulting in a final sample of 509 participants.

Among the 509 participants (*M* = 25.30, SD = 4.73) who were admitted to the FRITZ inpatient or day‐clinic treatment ward, 42.6% (*n* = 217) had a diagnosis of NSIPD, 33.3% (*n* = 173) had a diagnosis of CIPD, and 7% (*n* = 36) had a diagnosis of NSIPD+C. Most of the participants were male (67%) and had sought mental health treatment before being in the FRITZ ward (75.5%). See Table 2 for an overview of sociodemographic and clinical data.

**TABLE 2:** Sociodemographic and clinical descriptives of participants at baseline.

Sociodemographic and clinical characteristics	NSIPD sample (*n* = 217)		CIPD sample (*n* = 173)		NSIPD+C sample (*n* = 36)	
	*M*/*N*	SD/%	*M*/*N*	SD/%	*M*/*N*	SD/%
Age (years)	25.98	4.81	24.93	4.35	24.26	3.74
Gender (female)	79	36.4%	51	29.5%	11	30.6%
Marital status						
Single	185	85.3%	157	90.8%	33	91.7%
Education (years)	39	34.2	38			
Migration background (yes)	91	41.9%	69	39.9%	12	33.3%
Antipsychotics (yes)	167	77%	121	69.9%	31	86.1%
Monotherapy	141	65%	110	63.6%	23	63.9%
Mood stabilizers (yes)	17	7%	0	0	4	11%
GAF						
Admittance	39.70	14.25	40.25	14.73	38.61	12.63
Discharge	58.89	16.02	60.66	13.77	58.50	13.54
Length of treatment (days)	28.37	28.14	19.94	19.56		
Family history of psychosis (yes)	74	34.1%	45	26.0%	8	22.2%
DuP (days)	512.02	537.31	494.80	522.92	467.03	493.63
Insight into illness (no)						
Admittance	76	35%	46	26.6%	14	38.9%
Discharge	7	3.2%	4	2.3%	2	5.6%
Living situation						
Alone/family	134	61.8%	102	59%	16	44.4%
Homeless	14	6.5%	23	13.3%	7	19.4%
Working status^a^ (yes)	99	45.6%	63	36.4%	9	25%
Pathway to care						
Emergency room	26	12%	18	10.4%	7	19.4%
Inpatient locked unit	109	50.2%	110	63.6%	21	58.3%
Prior mental health treatment (yes)	156	71.9%	135	78%	31	86.1%

Abbreviations: DUP, duration of untreated psychosis; GAF, global assessment of functioning; M, mean; N, number; SD, standard deviation.

^a^ Full‐time Job or Studies.

### 3.1 Significant Outcomes

A Kruskal–Wallis H test indicated significant differences in length of treatment across diagnostic groups, *χ* ^2^(2) = 10.58, *p* < 0.01 (see Table 3). Mean rank scores were highest for the NSIPD group (229.28), followed by NSIPD+C (227.64) and CIPD (189.62), with higher ranks reflecting longer treatment durations. Post hoc Dunn tests with Bonferroni adjustment revealed that the NSIPD group had significantly longer treatment lengths than the CIPD group (*p* < 0.01). No significant differences emerged between NSIPD and NSIPD+C or between CIPD and NSIPD+C.

**TABLE 3:** Multivariate Kruskal–Wallis test and Dunn post hoc test results comparing continuous data characteristics across diagnoses.

Kruskal–Wallis test						Dunn post hoc comparison					
Variables	*N*	Mean rank	$\chi^{2}$	df	*p*	NSIPD vs. CIPD		NSIPD vs. NSIPD+C		CIPD vs. NSIPD+C	
						*Z*‐score	*p*	*Z*‐score	*p*	*Z*‐score	*p*
Length			10.58	2	< 0.01	1.64	0.01	0.45	0.99	1.04	0.23
NSIPD	217	229.28									
CIPD	173	189.62									
NSIPD+C	35	227.64									
Age			6.08	2	< 0.05	1.98	0.14	1.92	0.16	0.79	1.00
NSIPD	217	227.17									
CIPD	173	202.37									
NSIPD+C	36	184.56									
GAF			1.63	2	0.44						
NSIPD	193	189.68									
CIPD	154	192.30									
NSIPD+C	33	186.89									
DUP			1.07	2	0.59						
NSIPD	217	219.23									
CIPD	173	208.19									
NSIPD+C	36	204.49									
GAF diff			2.29	2	0.32						
SIPD	217	157.36									
CIPD	173	155.97									
NSIPD+C	36	128.40									

Abbreviations: CIPD, Cannabis‐induced psychotic disorder; df, degrees of freedom; DuP, duration of untreated psychosis; GAF, global assessment of functioning; GAF diff, change in GAF score (GAF at discharge‐GAF at admittance); *N*, number of participants; NSIPD, non‐substance induced psychotic disorder; NSIPD+C, non‐substance induced psychotic disorder with cannabis abuse.

Chi‐square tests of independence revealed significant associations between diagnostic groups and type of discharge, *χ* ^2^ = 8.44, 2 df, *p* = 0.01; therapy motivation, *χ* ^2^ = 10.72, 2 df, *p* = 0.01; and working status, *χ* ^2^ = 6.39, 2 df, *p* < 0.05 (see Table 4). Post hoc analyses indicated higher rates of clinician‐agreed discharges in the NSIPD group compared to CIPD and NSIPD+C groups. Similarly, patients in the NSIPD group exhibited higher rates of high therapy motivation relative to the other groups, whereas no significant group differences were observed for low/medium motivation levels. For working status, post hoc comparisons yielded no statistically significant pairwise associations.

**TABLE 4:** Group comparison between the NSIPD, CIPD and NSIPD+C (categorical data).

Characteristics	NSIPD, *n* (%)	CIPD, *n* (%)	NSIPD+C, *n* (%)	$\chi^{2}$	*p*	Cramer's υ
Gender (female)	79 (20.3)	51 (13.1)	11 (2.6)	2.20	0.33	0.07
Migration background (yes)	92 (23,8)	69 (17,9)	12 (2.9)	0.52	0.77	0.04
Type of discharge (in accordance with clinicians)	169 (40.3)	112 (26.7)	24 (5.7)	8.44	0.01	0.14
Insight into illness at admittance (yes)	47 (11.1)	39 (9.2)	6 (1.4)	0.52	0.80	0.04
Insight into illness at discharge (yes)	158 (37.9)	125 (30.0)	21 (5.0)	0.96	0.62	0.05
Therapy participation (yes)	161 (38.5)	116 (27.8)	27 (6.5)	4.76	0.09	0.11
Therapy motivation (high)	129 (30.9)	78 (18.7)	12 (2.9)	10.72	0.01	0.16
Prior treatment (yes)	156 (37.0)	135 (32.0)	31 (7.3)	5.45	0.07	0.11
Living status at discharge				11.18	0.08	0.12
Own household	64 (17.3)	51 (13.8)	10 (2.7)			
With Family	72 (19.5)	45 (12.2)	7 (1.9)			
Flatmates	31 (8.4)	37 (10.0)	5 (1.4)			
Homeless	17 (4.6)	24 (6.5)	7 (1.9)			
Working status (employed)	107 (28.7)	74 (19.8)	9 (2.4)	6.39	0.04	0.13
Family status (single)	184 (48.0)	157 (41.0)	33 (7.9)	4.51	0.11	0.11
Family history of psychosis (yes)	74 (22.3)	45 (13.6)	8 (2.4)	3.13	0.21	0.10
Antipsychotic treatment (yes)	167 (39.4)	121 (28.5)	31 (7.3)	5.57	0.06	0.12

Abbreviation: *n*, number of participants.

### 3.2 Nonsignificant Outcomes

A Kruskal–Wallis H test indicated that age did not differ significantly across groups, *χ* ^2^(2) = 6.08, *p* = 0.05. Mean rank age scores were highest for NSIPD (227.17), followed by CIPD (202.37) and NSIPD+C (184.56), with higher ranks denoting older age. No significant group differences were found for DUP or functioning (baseline GAF scores or pre‐to‐posttreatment GAF changes). No significant associations emerged between groups for disciplinary or against‐medical‐advice discharges.

## 4 Discussion

### 4.1 Main Findings

The aim of this study was to investigate sociodemographic and clinical characteristics of individuals with early psychosis, comparing CIPD, NSIPD+C, and NSIPD. Our results are the first to date examining clinical routine data from a clinical cohort that specifically met the criteria for early psychosis and was treated in a specialized early intervention center. With a large sample size, we deliberately chose to investigate three diagnostic groups to examine potential group differences in (a) primary psychosis versus induced psychosis and (b) the presence of cannabis use versus non‐use.

Participants with a diagnosis of NSIPD showed a significantly longer duration of inpatient treatment (LOS) compared to participants with CIPD and significantly higher rates of therapy motivation compared to both NSIPD+C and CIPD. Discharge in accordance with clinicians' recommendation was also significantly higher in the NSIPD group compared to both NSIPD+C and CIPD.

We did not find any group differences regarding level of functioning (GAF), age of onset, DUP, or further sociodemographic and clinical variables.

### 4.2 Interpretation of Findings and Comparison With Existing Literature

The lack of evidence of statistically significant group differences regarding most sociodemographic and clinical parameters when comparing NSIPD, NSIPD+C and CIPD further confirms the existing diagnostic dilemma in routine clinical practice in the case of the simultaneous occurrence of psychotic symptoms and cannabis use. Previous studies investigated group differences in NSIPD, NSIPD+C and CIPD and came to heterogeneous and partly discrepant findings regarding psychopathological parameters (Rentero et al. 2021; Rubio et al. 2012; Woolridge et al. 2023). The question therefore remains as to whether (a) we are talking about separate disease entities or a diagnostic continuum, and (b) whether we can and should make a clear distinction between NSIPD+C and CIPD in the early stage of a psychotic disorder.

National and international early intervention guidelines unanimously recommend that patients with a first episode of psychosis (FEP) should be treated in a specific early intervention setting regardless of a diagnosis of primary psychosis or substance induced psychosis (Early Psychosis Guidelines Writing Group 2016; Psychiatrists 2018). These recommendations are partly based on the continuing challenge in the diagnostic classification when cannabis use and psychotic symptoms simultaneously occur, and high transition rates of CIPD into a primary psychotic disorder within the first 3–5 years of illness (Starzer et al. 2018). Previous studies demonstrate clear evidence for better outcomes across a variety of parameters for treatment in specialized EIS compared to standard treatment (TAU) (Solmi et al. 2023) also for patients with comorbid cannabis use (Archie et al. 2007; Wright et al. 2023).

### 4.3 Length of Stay and DUP

Our results of longer LOS in participants with NSIPD compared to CIPD are in line with previous findings on the impact of substance use and dual diagnosis on LOS (Gomez‐Sanchez‐Lafuente et al. 2022). However, the lack of statistical significance in comparison with the NSIPD+C group raises the question of the extent to which cannabis use has a direct impact on treatment duration and reinforces that existing cannabis use is not a suitable predictor of LOS (Williams et al. 2021). Participants with NSIPD were more likely to be discharged in accordance with clinicians' recommendations; however, we did not find group differences regarding disciplinary discharge, so premature treatment discontinuations cannot be used for causal explanation for shorter LOS in the CIPD group. Previous studies have shown that longer DUP and symptom severity correlate with longer LOS (DGPPN 2019). In the present study, we could not detect significant group differences in DUP, which is in contrast to previous findings in this population of longer DUP in cannabis users versus non‐users (Schimmelmann et al. 2012; Seddon et al. 2016) and may reflect the efficacy of the low‐threshold and multidisciplinary access provided by FRITZ. Prior studies have shown that (i) implementation of early intervention strategies (EIS) can have a positive impact on DUP (Salazar de Pablo et al. 2024) and (ii) social networks supporting engagement with EIS further reduce DUP also in the presence of comorbid cannabis use (Carra et al. 2018).

### 4.4 Level of Functioning

Despite the widely reported negative impact of cannabis use on the course of a psychotic disorder (Hasan et al. 2020; Marconi et al. 2016; Schoeler et al. 2016), the influence of cannabis use on the level of functioning at the first manifestation of psychotic symptoms appears complex. While some studies demonstrated that patients with FEP who used cannabis had better functioning on several cognitive domains compared to FEP who were not cannabis users (Yucel et al. 2012), other studies report poorer psychosocial functioning in cannabis users compared to non‐users, especially when cannabis use is continued after diagnosis of FEP (Seddon et al. 2016). In the present study, we did not find group differences regarding the level of social functioning measured by GAF.

## 5 Limitations

There are several limitations to the present study. First, diagnoses of NSIPD, NSIPD+C, and CIPD were based on ICD‐10, which requires abstinence from cannabis for a minimum of 6 months for the diagnosis of primary psychoses, whereas DSM‐5 requires abstinence for 30 days. Our results of no statistical group differences between NSIPD+C and CIPD could partly be explained by this, as the CIPD group may have included participants who already fulfil the criteria of a primary psychosis according to DSM‐5, which also makes a comparison with previous studies on this topic more difficult. Nevertheless, as ICD‐10 is widely used and well accepted, this reflects the current diagnostic dilemma in routine clinical practice. Since the present study is a retrospective evaluation of routine clinical data, it was not possible to review the diagnoses according to DSM‐5.

Second, the routine clinical data examined did not contain detailed information on psychopathology, cannabis use parameters, and cognitive functioning. The evaluation of symptom severity was based on the external assessment of the clinical global impression (CGI) (Guy 1976). Due to the retrospective study design, it was not possible to retrospectively collect additional parameters. Cognitive impairment is common in schizophrenia; however, it may not present in all patients, particularly in the early stages of the disease. Furthermore, the NSIPD cohorts in this study also included individuals with a diagnosis of affective psychosis, who may not exhibit cognitive dysfunction (Zanelli et al. 2010). Previous research has shown that schizophreniform and affective psychoses have a high genetic heritability and an overlapping genetic risk in common (Stahl et al. 2019). We deliberately opted for the inclusion criteria mentioned since relevant diagnostic instability regarding the transition to manifest schizophrenia has been demonstrated in the early stages of a psychotic disorder (Fusar‐Poli et al. 2016).

Lastly, the NSIPD+C cohort presented with a smaller sample size compared to both NSIPD and CIPD cohorts, which may limit statistical power.

## 6 Implications for Future Research and Clinical Practice

Despite the convincing evidence for the effectiveness of EIS in early psychosis, also in patients who actively use cannabis, such services have not yet been implemented across all mental health services around the world. In addition, active substance use continues to be a frequent exclusion criterion in studies on early detection and treatment of psychotic disorders, while in clinical practice the co‐occurrence of psychotic symptoms and cannabis use is rather more common than exceptional. Since there are no known diagnostic parameters to date for clear differentiation between CIPD and NSIPD+C, further studies on the early detection and intervention of psychotic disorders should therefore include participants with active cannabis use to refine diagnostic assessments and treatment recommendations. Regarding clinical practice, an integrative approach addressing the treatment of psychosis and substance use concurrently and in a multidisciplinary manner should be pursued.

Due to several limitations, we could not examine variables according to DSM‐5 diagnoses of NSIPD, NSIPD+C, and CIPD. Further studies should address temporal differences in current diagnostic classification systems and employ a longitudinal design to examine not just predictive parameters for diagnosis, but also for transition from CIPD to primary psychosis.

Finally, a detailed assessment of cannabis use patterns seems highly relevant when investigating cannabis‐related (mental) health outcomes, especially regarding frequency, amount, and age of onset of cannabis use. To increase statistical power and confirm our findings, future studies should use a larger sample size of NSIPD+C when comparing NSIPD+C, CIPD, and NSIPD.

## Funding

The authors have nothing to report.

## Conflicts of Interest

The authors declare no conflicts of interest.

## Data Availability

The data that support the findings of this study are available from the corresponding author upon reasonable request. Analytic code supporting the findings is available from LH upon reasonable request. There are no plans to make further material, such as CRFs, available to other researchers.
